# Crystal structure of (*E*)-4-hy­droxy-*N*′-(3-hy­droxy­benzyl­idene)benzohydrazide monohydrate

**DOI:** 10.1107/S1600536814011908

**Published:** 2014-08-01

**Authors:** William T. A. Harrison, John Nicolson Low, James L. Wardell

**Affiliations:** a Department of Chemistry, University of Aberdeen, Meston Walk, Aberdeen AB24 3UE, Scotland; b FioCruz-Fundação Oswaldo Cruz, Instituto de Tecnologia em Fármacos-Far-Manguinhos, Rua Sizenando Nabuco, 100, Manguinhos, 21041-250 Rio de Janeiro, RJ, Brazil

**Keywords:** crystal structure, benzohydrazide, hydrate, hydrogen bonding

## Abstract

In the title benzohydrazide hydrate, C_14_H_12_N_2_O_3_·H_2_O, the dihedral angle between the aromatic rings is 58.11 (6)° and the C=O and N—H groups adopt an *anti* orientation. The main twist in the mol­ecule occurs about the C(=O)—C_ar_ (ar = aromatic) bond, with an N—C(=O)—C_ar_—C_ar_ torsion angle of −43.5 (2)°. In the crystal, the components are linked by N—H⋯O, O—H⋯N and O—H⋯O hydrogen bonds. These inter­actions generate [10-1] chains, with adjacent organic mol­ecules linked by inversion symmetry generating either pairs of N—H⋯O links [*R*_2_^2^(16) loops] or pairs of O—H⋯O links [*R*_2_^2^(20) loops]. Pairs of water mol­ecules are located in the *R*_2_^2^(20) loops and form their own O—H⋯O and O—H⋯N hydrogen bonds to adjacent organic mol­ecules in the chain. Finally, an inter­chain O—H⋯O hydrogen-bond link from the 4-hy­droxy group generates (010) sheets.

## Related literature   

For a related structure, see: Fun *et al.* (2011 ▶). A survey of the Cambridge Structural Database (Version 5.35 of November 2013; Allen, 2002 ▶) revealed no fewer than 581 distinct benzohydrazide fragments with different substituents on the aromatic rings and/or other chemical species in the crystal: all bond lengths for the central fragment of the title compound lie close to the mean values for these structures. The only parameter in the metrical survey that shows significant variation is the dihedral angle between the aromatic rings, with the most common value close to zero, and a roughly linear decrease to 90°.
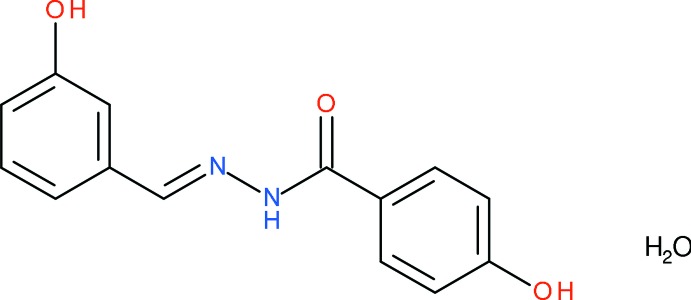


## Experimental   

### Crystal data  

C_14_H_12_N_2_O_3_·H_2_O
*M* *_r_* = 274.27
Triclinic, 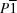

*a* = 7.1826 (5) Å
*b* = 9.2043 (6) Å
*c* = 10.7568 (7) Å
α = 91.847 (7)°
β = 102.433 (7)°
γ = 110.191 (8)°
*V* = 647.37 (7) Å^3^
*Z* = 2
Mo *K*α radiation
μ = 0.11 mm^−1^
*T* = 100 K
0.09 × 0.05 × 0.02 mm

### Data collection  

Rigaku Saturn CCD diffractometer
8728 measured reflections
2962 independent reflections
2038 reflections with *I* > 2σ(*I*)
*R*_int_ = 0.049

### Refinement  

*R*[*F* ^2^ > 2σ(*F* ^2^)] = 0.044
*wR*(*F* ^2^) = 0.105
*S* = 1.06
2962 reflections
196 parameters
H atoms treated by a mixture of independent and constrained refinement
Δρ_max_ = 0.27 e Å^−3^
Δρ_min_ = −0.28 e Å^−3^

### 

Data collection: *CrystalClear* (Rigaku, 2010 ▶); cell refinement: *CrystalClear*; data reduction: *CrystalClear*; program(s) used to solve structure: *SHELXS97* (Sheldrick, 2008 ▶); program(s) used to refine structure: *SHELXL97* (Sheldrick, 2008 ▶); molecular graphics: *ORTEP-3 for Windows* (Farrugia, 2012 ▶); software used to prepare material for publication: *SHELXL97*.

## Supplementary Material

Crystal structure: contains datablock(s) I, global. DOI: 10.1107/S1600536814011908/su0006sup1.cif

Structure factors: contains datablock(s) I. DOI: 10.1107/S1600536814011908/su0006Isup2.hkl

Supporting information file. DOI: 10.1107/S1600536814011908/su0006Isup3.cml

**Fig. 1.**
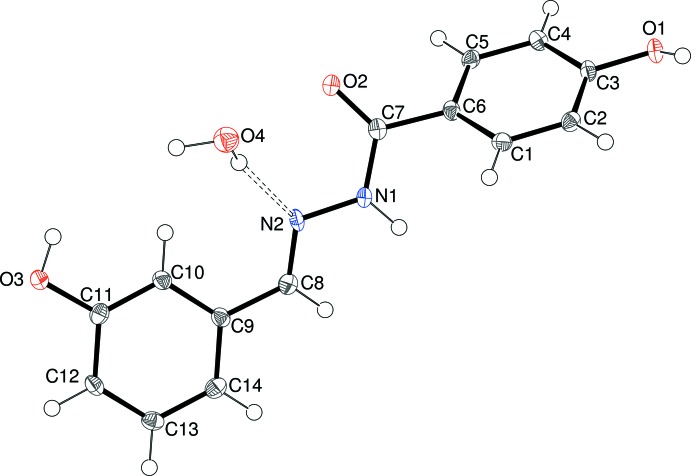
A view of the asymmetric unit of the title compound, showing the atom-labelling scheme. Displacement ellipsoids are drawn at the 50% probability level.

. DOI: 10.1107/S1600536814011908/su0006fig1.tif
A view of the asymmetric unit of the title compound, showing the atom-labelling scheme. Displacement ellipsoids are drawn at the 50% probability level.

**Fig. 2.**
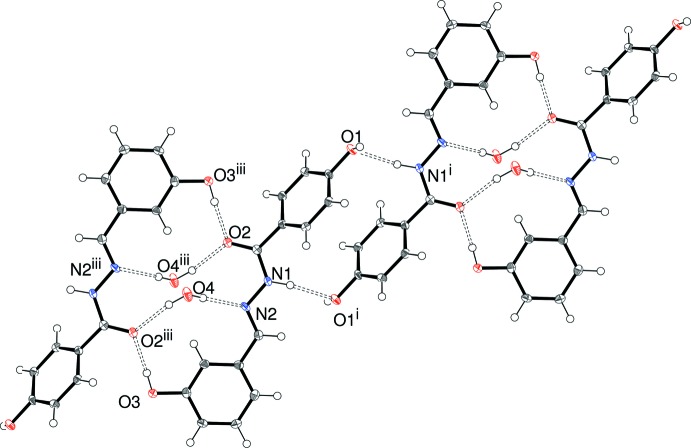
A fragment of a [101] chain in the structure of the title compound, with hydrogen bonds shown as double-dashed lines, showing the R22(16) loops and R22(20) loops between adjacent organic molecules (see Table 2 for details of the hydrogen bonding and the symmetry codes).

. DOI: 10.1107/S1600536814011908/su0006fig2.tif
A fragment of a [10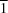] chain in the structure of the title compound, with hydrogen bonds shown as double-dashed lines, showing the 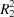
(16) loops and 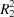
(20) loops between adjacent organic mol­ecules (see Table 2 for details of the hydrogen bonding and the symmetry codes).

**Fig. 3.**
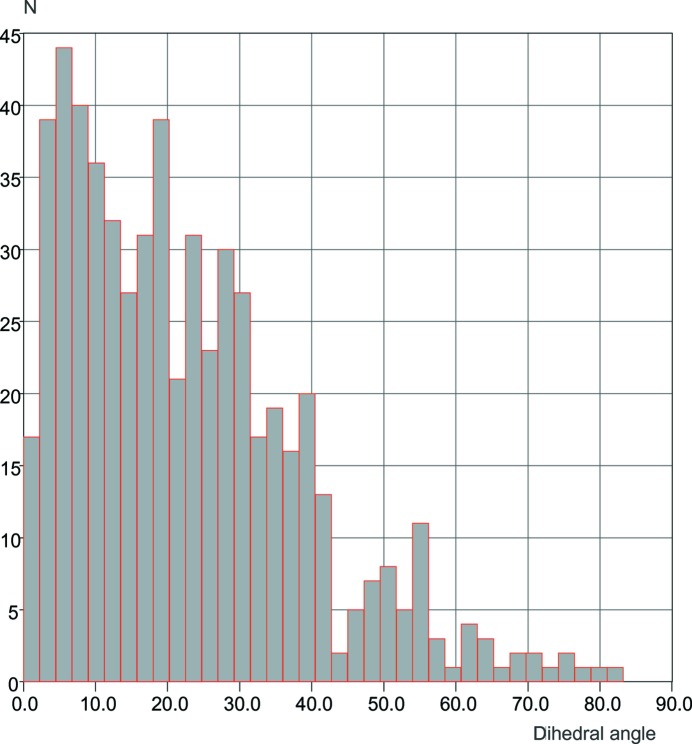
A histogram of dihedral angles between the aromatic rings of benzohydrazide structures in the Cambridge Structural Database (Version 5.35; Allen, 2002).

. DOI: 10.1107/S1600536814011908/su0006fig3.tif
A histogram of dihedral angles between the aromatic rings of benzohydrazide structures in the Cambridge Structural Database (Version 5.35; Allen, 2002).

CCDC reference: 1004470

Additional supporting information:  crystallographic information; 3D view; checkCIF report

## Figures and Tables

**Table 1:** Hydrogen-bond geometry (Å, °)

*D*—H⋯*A*	*D*—H	H⋯*A*	*D*⋯*A*	*D*—H⋯*A*
N1—H1*N*⋯O1^i^	0.922 (18)	2.044 (19)	2.9357 (19)	162.2 (16)
O1—H1*O*⋯O4^ii^	0.92 (2)	1.68 (2)	2.6025 (18)	172.6 (17)
O3—H3*O*⋯O2^iii^	0.92 (2)	1.83 (2)	2.7512 (17)	178.1 (17)
O4—H1*W*⋯N2	0.86 (2)	2.16 (2)	3.018 (2)	172.5 (19)
O4—H2*W*⋯O2^iii^	0.89 (2)	1.98 (2)	2.8676 (17)	170.5 (18)

Symmetry codes: (i) 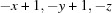; (ii) 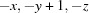; (iii) 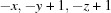.

## supplementary crystallographic information

### S1. Synthesis and crystallization

Equimolar qu­anti­ties of 4-hy­droxy­benzohydrazide and 3-hy­droxy­benzaldehyde were refluxed in ethanol for several hours and then cooled to room temperature. Colourless chips of the title compound were obtained by slow evaporation of the solvent at room temperature after several days.

### S2. Refinement

O- and N-bound H atoms were located in difference Fourier maps and their positions were freely refined. C-bound H atoms were placed in idealized positions (C—H = 0.93 Å) and refined as riding atoms. The constraint *U*_iso_(H) = 1.2*U*_eq_(carrier) was applied in all cases.

### Crystal data

**Table:**

C_14_H_12_N_2_O_3_·H_2_O	*Z* = 2
*M**_r_* = 274.27	*F*(000) = 288
Triclinic, *P*1	*D*_x_ = 1.407 Mg m^−^^3^
Hall symbol: -P 1	Mo *K*α radiation, λ = 0.71073 Å
*a* = 7.1826 (5) Å	Cell parameters from 6867 reflections
*b* = 9.2043 (6) Å	θ = 3.1–27.5°
*c* = 10.7568 (7) Å	µ = 0.11 mm^−^^1^
α = 91.847 (7)°	*T* = 100 K
β = 102.433 (7)°	Chip, colourless
γ = 110.191 (8)°	0.09 × 0.05 × 0.02 mm
*V* = 647.37 (7) Å^3^	

### Data collection

**Table:**

Rigaku Saturn CCD diffractometer	2038 reflections with *I* > 2σ(*I*)
Radiation source: fine-focus sealed tube	*R*_int_ = 0.049
Graphite monochromator	θ_max_ = 27.5°, θ_min_ = 3.1°
ω scans	*h* = −9→9
8728 measured reflections	*k* = −11→11
2962 independent reflections	*l* = −13→13

### Refinement

**Table:**

Refinement on *F*^2^	Primary atom site location: structure-invariant direct methods
Least-squares matrix: full	Secondary atom site location: difference Fourier map
*R*[*F*^2^ > 2σ(*F*^2^)] = 0.044	Hydrogen site location: inferred from neighbouring sites
*wR*(*F*^2^) = 0.105	H atoms treated by a mixture of independent and constrained refinement
*S* = 1.06	*w* = 1/[σ^2^(*F*_o_^2^) + (0.0374*P*)^2^ + 0.144*P*] where *P* = (*F*_o_^2^ + 2*F*_c_^2^)/3
2962 reflections	(Δ/σ)_max_ < 0.001
196 parameters	Δρ_max_ = 0.27 e Å^−^^3^
0 restraints	Δρ_min_ = −0.28 e Å^−^^3^

### Special details

**Table:**

Geometry. All e.s.d.'s (except the e.s.d. in the dihedral angle between two l.s. planes) are estimated using the full covariance matrix. The cell e.s.d.'s are taken into account individually in the estimation of e.s.d.'s in distances, angles and torsion angles; correlations between e.s.d.'s in cell parameters are only used when they are defined by crystal symmetry. An approximate (isotropic) treatment of cell e.s.d.'s is used for estimating e.s.d.'s involving l.s. planes.
Refinement. Refinement of *F*^2^ against ALL reflections. The weighted *R*-factor *wR* and goodness of fit *S* are based on *F*^2^, conventional *R*-factors *R* are based on *F*, with *F* set to zero for negative *F*^2^. The threshold expression of *F*^2^ > σ(*F*^2^) is used only for calculating *R*-factors(gt) *etc*. and is not relevant to the choice of reflections for refinement. *R*-factors based on *F*^2^ are statistically about twice as large as those based on *F*, and *R*- factors based on ALL data will be even larger.

### Fractional atomic coordinates and isotropic or equivalent isotropic displacement parameters (Å^2^)

**Table:**

	*x*	*y*	*z*	*U*_iso_*/*U*_eq_	
C1	0.2487 (2)	0.51249 (18)	0.02953 (16)	0.0151 (4)	
H1	0.2596	0.6145	0.0499	0.018*	
C2	0.2488 (2)	0.46303 (18)	−0.09366 (16)	0.0149 (4)	
H2	0.2560	0.5306	−0.1564	0.018*	
C3	0.2379 (2)	0.31187 (19)	−0.12226 (15)	0.0140 (4)	
C4	0.2225 (2)	0.20863 (19)	−0.02998 (16)	0.0150 (4)	
H4	0.2163	0.1077	−0.0499	0.018*	
C5	0.2167 (2)	0.25738 (18)	0.09144 (16)	0.0145 (4)	
H5	0.2022	0.1879	0.1527	0.017*	
C6	0.2325 (2)	0.41045 (18)	0.12303 (15)	0.0133 (4)	
C7	0.2265 (3)	0.45636 (18)	0.25514 (16)	0.0140 (4)	
C8	0.5163 (3)	0.77584 (19)	0.48276 (16)	0.0164 (4)	
H8	0.6243	0.8109	0.4432	0.020*	
C9	0.5316 (3)	0.85792 (18)	0.60520 (16)	0.0145 (4)	
C10	0.3753 (3)	0.80721 (18)	0.66983 (16)	0.0148 (4)	
H10	0.2637	0.7159	0.6379	0.018*	
C11	0.3865 (3)	0.89243 (18)	0.78093 (16)	0.0154 (4)	
C12	0.5575 (3)	1.02744 (18)	0.83137 (16)	0.0158 (4)	
H12	0.5659	1.0846	0.9066	0.019*	
C13	0.7134 (3)	1.07503 (18)	0.76867 (16)	0.0162 (4)	
H13	0.8278	1.1638	0.8029	0.019*	
C14	0.7017 (3)	0.99231 (18)	0.65541 (16)	0.0167 (4)	
H14	0.8066	1.0262	0.6132	0.020*	
N1	0.3623 (2)	0.59830 (16)	0.30909 (13)	0.0156 (3)	
H1N	0.469 (3)	0.647 (2)	0.2723 (17)	0.019*	
N2	0.3584 (2)	0.65697 (15)	0.42830 (13)	0.0153 (3)	
O1	0.24457 (19)	0.25851 (13)	−0.24143 (11)	0.0173 (3)	
H1O	0.195 (3)	0.309 (2)	−0.3059 (19)	0.021*	
O2	0.10482 (18)	0.37043 (13)	0.31151 (11)	0.0177 (3)	
O3	0.23698 (19)	0.84893 (14)	0.84718 (12)	0.0198 (3)	
H3O	0.125 (3)	0.776 (2)	0.7930 (18)	0.024*	
O4	−0.0770 (2)	0.61780 (16)	0.42664 (13)	0.0236 (3)	
H1W	0.047 (3)	0.624 (2)	0.4329 (19)	0.028*	
H2W	−0.101 (3)	0.615 (2)	0.505 (2)	0.028*	

### Atomic displacement parameters (Å^2^)

**Table:**

	*U*^11^	*U*^22^	*U*^33^	*U*^12^	*U*^13^	*U*^23^
C1	0.0145 (8)	0.0140 (8)	0.0159 (9)	0.0042 (7)	0.0031 (7)	0.0012 (7)
C2	0.0147 (9)	0.0163 (8)	0.0138 (9)	0.0055 (7)	0.0035 (7)	0.0049 (7)
C3	0.0122 (8)	0.0206 (8)	0.0095 (8)	0.0067 (7)	0.0024 (6)	0.0007 (7)
C4	0.0157 (9)	0.0157 (8)	0.0140 (9)	0.0073 (7)	0.0021 (7)	0.0004 (7)
C5	0.0147 (8)	0.0154 (8)	0.0127 (9)	0.0047 (7)	0.0028 (7)	0.0046 (7)
C6	0.0117 (8)	0.0173 (8)	0.0111 (8)	0.0056 (7)	0.0021 (6)	0.0014 (6)
C7	0.0149 (8)	0.0163 (8)	0.0129 (9)	0.0081 (7)	0.0029 (7)	0.0024 (7)
C8	0.0164 (9)	0.0175 (8)	0.0164 (9)	0.0062 (7)	0.0057 (7)	0.0025 (7)
C9	0.0171 (9)	0.0139 (8)	0.0116 (9)	0.0058 (7)	0.0017 (7)	0.0015 (6)
C10	0.0164 (9)	0.0138 (8)	0.0124 (9)	0.0040 (7)	0.0019 (7)	0.0011 (7)
C11	0.0166 (9)	0.0168 (8)	0.0150 (9)	0.0076 (7)	0.0050 (7)	0.0048 (7)
C12	0.0194 (9)	0.0151 (8)	0.0120 (9)	0.0067 (7)	0.0014 (7)	−0.0009 (7)
C13	0.0159 (9)	0.0133 (8)	0.0167 (9)	0.0042 (7)	0.0001 (7)	0.0017 (7)
C14	0.0155 (9)	0.0174 (8)	0.0176 (9)	0.0062 (7)	0.0041 (7)	0.0055 (7)
N1	0.0175 (8)	0.0176 (7)	0.0117 (7)	0.0043 (6)	0.0071 (6)	0.0008 (6)
N2	0.0207 (8)	0.0176 (7)	0.0091 (7)	0.0081 (6)	0.0051 (6)	−0.0002 (6)
O1	0.0219 (7)	0.0225 (6)	0.0098 (6)	0.0104 (5)	0.0044 (5)	0.0018 (5)
O2	0.0195 (7)	0.0190 (6)	0.0136 (6)	0.0039 (5)	0.0068 (5)	0.0010 (5)
O3	0.0181 (7)	0.0203 (6)	0.0164 (7)	0.0001 (5)	0.0070 (5)	−0.0041 (5)
O4	0.0222 (7)	0.0391 (8)	0.0145 (7)	0.0154 (6)	0.0069 (6)	0.0081 (6)

### Geometric parameters (Å, º)

**Table:**

C1—C2	1.387 (2)	C9—C14	1.394 (2)
C1—C6	1.394 (2)	C9—C10	1.399 (2)
C1—H1	0.9300	C10—C11	1.380 (2)
C2—C3	1.387 (2)	C10—H10	0.9300
C2—H2	0.9300	C11—O3	1.3685 (19)
C3—O1	1.3743 (19)	C11—C12	1.401 (2)
C3—C4	1.391 (2)	C12—C13	1.381 (2)
C4—C5	1.381 (2)	C12—H12	0.9300
C4—H4	0.9300	C13—C14	1.387 (2)
C5—C6	1.398 (2)	C13—H13	0.9300
C5—H5	0.9300	C14—H14	0.9300
C6—C7	1.483 (2)	N1—N2	1.3847 (18)
C7—O2	1.2404 (19)	N1—H1N	0.922 (18)
C7—N1	1.350 (2)	O1—H1O	0.92 (2)
C8—N2	1.281 (2)	O3—H3O	0.92 (2)
C8—C9	1.461 (2)	O4—H1W	0.86 (2)
C8—H8	0.9300	O4—H2W	0.89 (2)
			
C2—C1—C6	120.52 (15)	C14—C9—C8	119.08 (15)
C2—C1—H1	119.7	C10—C9—C8	121.08 (14)
C6—C1—H1	119.7	C11—C10—C9	120.12 (15)
C3—C2—C1	119.34 (15)	C11—C10—H10	119.9
C3—C2—H2	120.3	C9—C10—H10	119.9
C1—C2—H2	120.3	O3—C11—C10	122.49 (15)
O1—C3—C2	121.75 (15)	O3—C11—C12	117.45 (15)
O1—C3—C4	117.34 (15)	C10—C11—C12	120.05 (15)
C2—C3—C4	120.90 (15)	C13—C12—C11	119.56 (15)
C5—C4—C3	119.41 (15)	C13—C12—H12	120.2
C5—C4—H4	120.3	C11—C12—H12	120.2
C3—C4—H4	120.3	C12—C13—C14	120.89 (15)
C4—C5—C6	120.53 (15)	C12—C13—H13	119.6
C4—C5—H5	119.7	C14—C13—H13	119.6
C6—C5—H5	119.7	C13—C14—C9	119.56 (15)
C1—C6—C5	119.25 (15)	C13—C14—H14	120.2
C1—C6—C7	122.63 (15)	C9—C14—H14	120.2
C5—C6—C7	118.11 (15)	C7—N1—N2	119.60 (14)
O2—C7—N1	122.60 (15)	C7—N1—H1N	119.1 (11)
O2—C7—C6	122.25 (14)	N2—N1—H1N	120.5 (11)
N1—C7—C6	115.15 (14)	C8—N2—N1	114.82 (14)
N2—C8—C9	121.91 (15)	C3—O1—H1O	112.8 (11)
N2—C8—H8	119.0	C11—O3—H3O	106.5 (11)
C9—C8—H8	119.0	H1W—O4—H2W	108.6 (18)
C14—C9—C10	119.79 (15)		
			
C6—C1—C2—C3	−1.7 (2)	N2—C8—C9—C10	1.6 (3)
C1—C2—C3—O1	−177.71 (15)	C14—C9—C10—C11	1.9 (3)
C1—C2—C3—C4	1.4 (2)	C8—C9—C10—C11	−175.61 (16)
O1—C3—C4—C5	179.58 (14)	C9—C10—C11—O3	179.33 (16)
C2—C3—C4—C5	0.4 (2)	C9—C10—C11—C12	−1.9 (3)
C3—C4—C5—C6	−2.0 (2)	O3—C11—C12—C13	179.29 (16)
C2—C1—C6—C5	0.2 (2)	C10—C11—C12—C13	0.5 (3)
C2—C1—C6—C7	−178.43 (15)	C11—C12—C13—C14	1.0 (3)
C4—C5—C6—C1	1.7 (2)	C12—C13—C14—C9	−1.0 (3)
C4—C5—C6—C7	−179.64 (15)	C10—C9—C14—C13	−0.4 (3)
C1—C6—C7—O2	136.31 (18)	C8—C9—C14—C13	177.13 (15)
C5—C6—C7—O2	−42.3 (2)	O2—C7—N1—N2	−4.7 (3)
C1—C6—C7—N1	−43.5 (2)	C6—C7—N1—N2	175.08 (14)
C5—C6—C7—N1	137.90 (16)	C9—C8—N2—N1	176.21 (15)
N2—C8—C9—C14	−175.95 (16)	C7—N1—N2—C8	166.42 (16)

### Hydrogen-bond geometry (Å, º)

**Table:**

*D*—H···*A*	*D*—H	H···*A*	*D*···*A*	*D*—H···*A*
N1—H1*N*···O1^i^	0.922 (18)	2.044 (19)	2.9357 (19)	162.2 (16)
O1—H1*O*···O4^ii^	0.92 (2)	1.68 (2)	2.6025 (18)	172.6 (17)
O3—H3*O*···O2^iii^	0.92 (2)	1.83 (2)	2.7512 (17)	178.1 (17)
O4—H1*W*···N2	0.86 (2)	2.16 (2)	3.018 (2)	172.5 (19)
O4—H2*W*···O2^iii^	0.89 (2)	1.98 (2)	2.8676 (17)	170.5 (18)

Symmetry codes: (i) −*x*+1, −*y*+1, −*z*; (ii) −*x*, −*y*+1, −*z*; (iii) −*x*, −*y*+1, −*z*+1.
