# Supplementary figures and images for: Crystal structure of (E)-4-hy­droxy-N′-(3-hy­droxy­benzyl­idene)benzohydrazide monohydrate
Source: Acta Crystallogr Sect E Struct Rep Online. 2014 Aug 1;70(Pt 9):o891–2. doi: 10.1107/S1600536814011908 (PMC4186143; doi:10.1107/S1600536814011908)

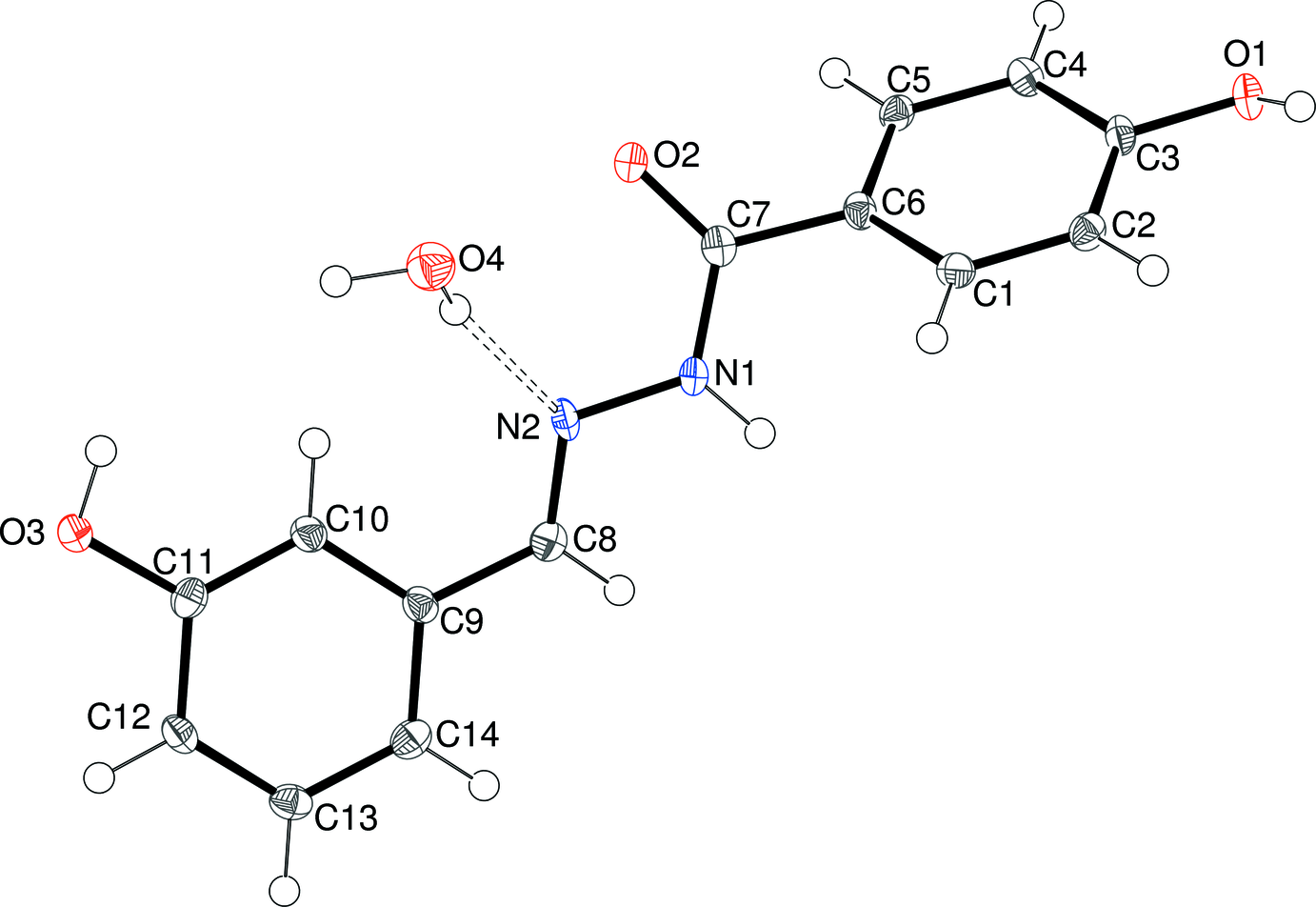

Supplement: Supplementary file 4 [file e-70-0o891-fig1.tif]

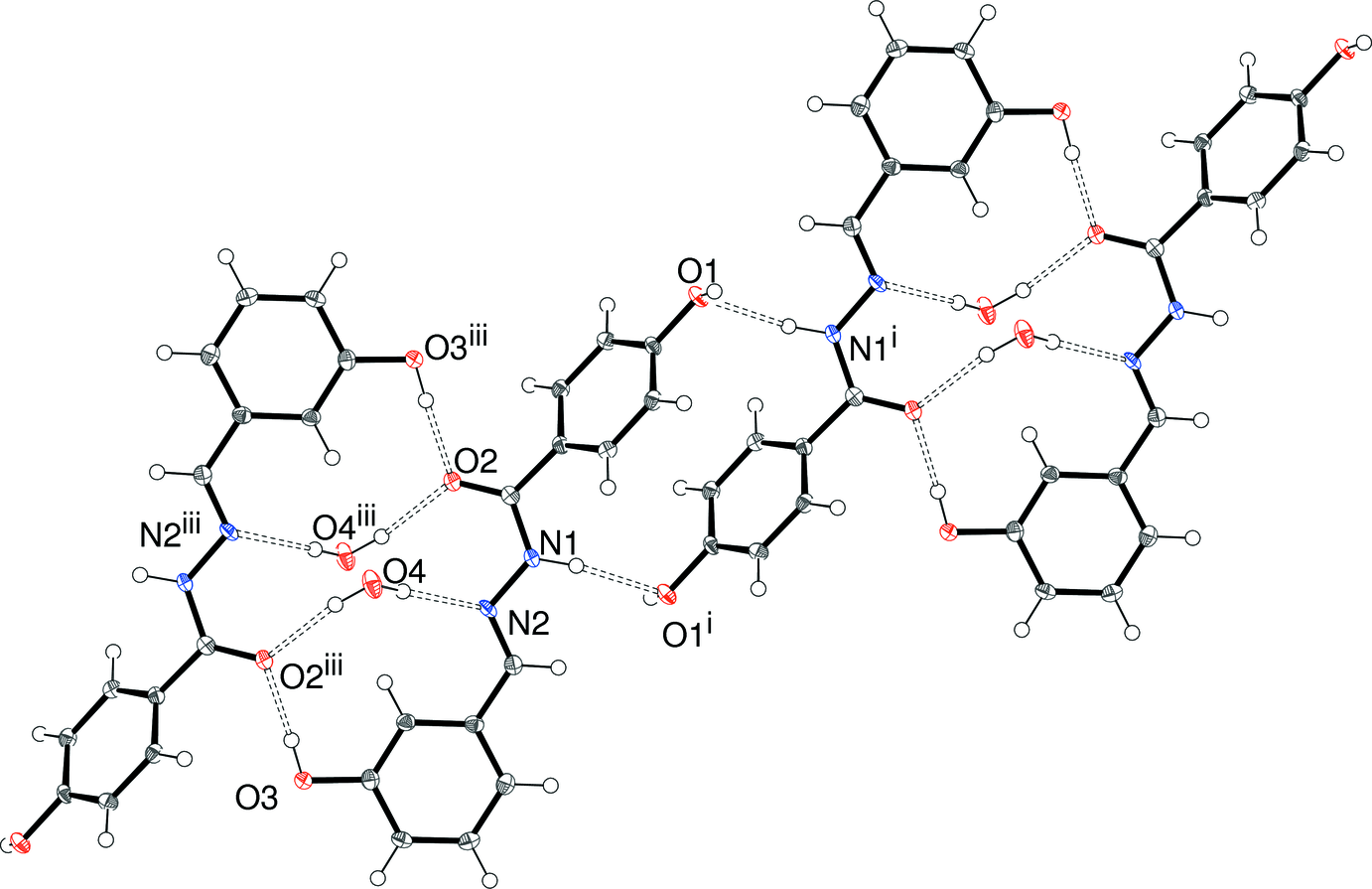

Supplement: Supplementary file 5 [file e-70-0o891-fig2.tif]

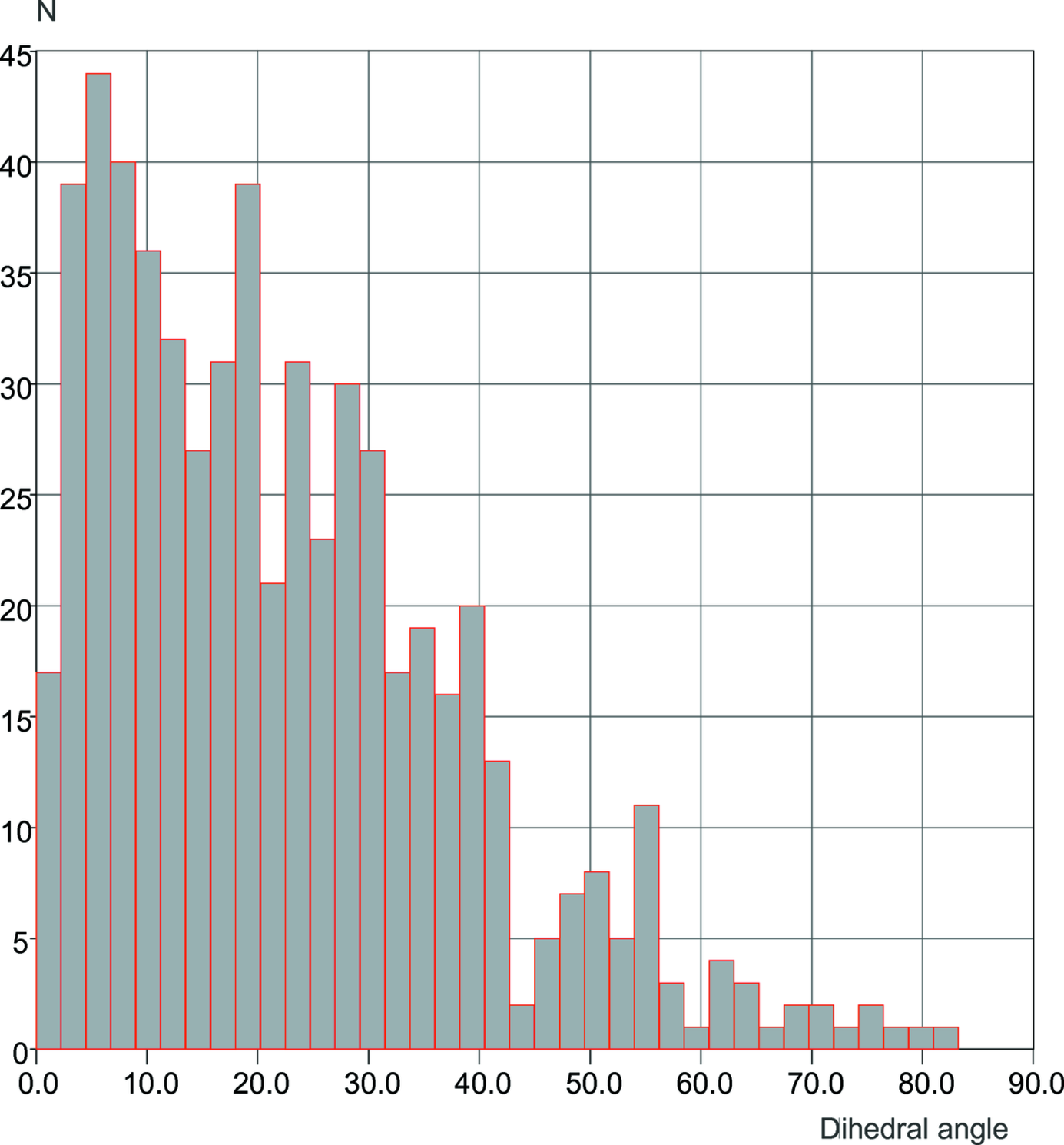

Supplement: Supplementary file 6 [file e-70-0o891-fig3.tif]
